# 223. Safety, Tolerability, and Immunogenicity of the mRNA-1345 RSV Vaccine in Solid Organ Transplant Recipients Aged ≥18 Years

**DOI:** 10.1093/ofid/ofaf695.081

**Published:** 2026-01-11

**Authors:** Erick F Mayer, Ann R Falsey, Cameron R Wolfe, Erica Herc, Fiona Burns, Dima Kabbani, Deepali Kumar, Christina Grassi, Joseph Whitten, Anthony Rizk, Caroline Reuter, Avi Collins, Anisha Mannan, Archana Kapoor, Sonia K Stoszek, Jiejun Du, Jenni Mou, Lan Lan, Honghong Zhou, Eleanor Wilson, Jaya Gowami, Rituparna Das, Frances Priddy

**Affiliations:** Moderna, Inc., Cambridge, MA; University of Rochester School of Medicine, Rochester, New York; Duke University, Durham, NC; Henry Ford Hospital, Detroit, Michigan; University College London, London, England, United Kingdom; University of Alberta, Edmonton, AB, Canada; University Of Toronto, Toronto, ON, Canada; Moderna, Inc., Cambridge, MA; Moderna, Inc., Cambridge, MA; Moderna, Inc., Cambridge, MA; Moderna, Inc., Cambridge, MA; Moderna, Inc., Cambridge, MA; Moderna, Inc., Cambridge, MA; Moderna, Inc., Cambridge, MA; Moderna, Inc., Cambridge, MA; Moderna, Inc., Cambridge, MA; Moderna, Inc., Cambridge, MA; Moderna, Inc., Cambridge, MA; Moderna, Inc., Cambridge, MA; Moderna, Inc., Cambridge, MA; Moderna, Inc., Cambridge, MA; Moderna, Inc., Cambridge, MA; Moderna, Inc., Cambridge, MA

## Abstract

**Background:**

Solid organ transplant recipients (SOTRs) are at increased risk for severe respiratory syncytial virus (RSV) disease due to chronic immunosuppression. Interim safety and immunogenicity are presented from a phase 3 trial evaluating 2 mRNA-1345 doses in SOTRs ≥18 years.

**Figure 1.**
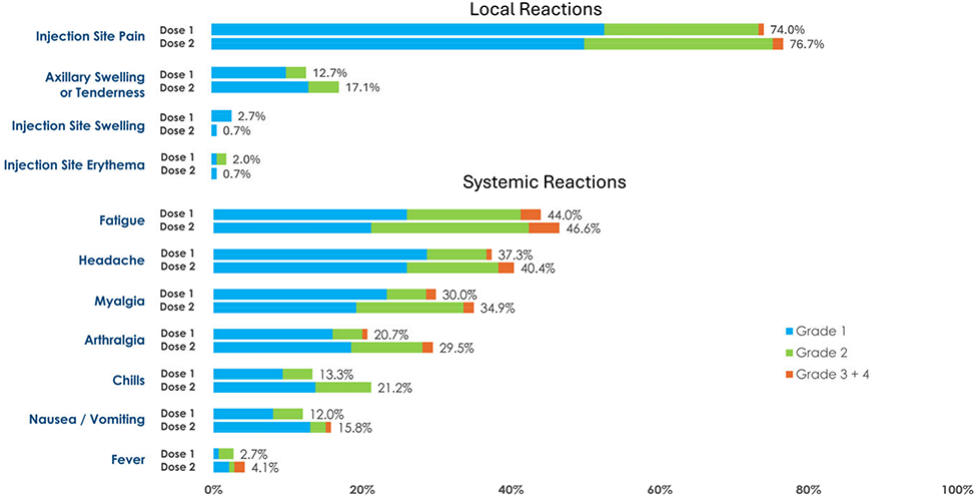
Solicited Local and Systemic Reactions within 7 Days After the First and Second Injections (Solicited Safety Set) Dose 1, N = 150; Dose 2, N = 146. Percentages are based on the number of exposed participants who submitted any data for the event. No grade 4 solicited adverse reactions were reported besides a single grade 4 systemic reaction of arthralgia reported for 1 participant (0.7%) after Dose 2.

**Figure 2.**
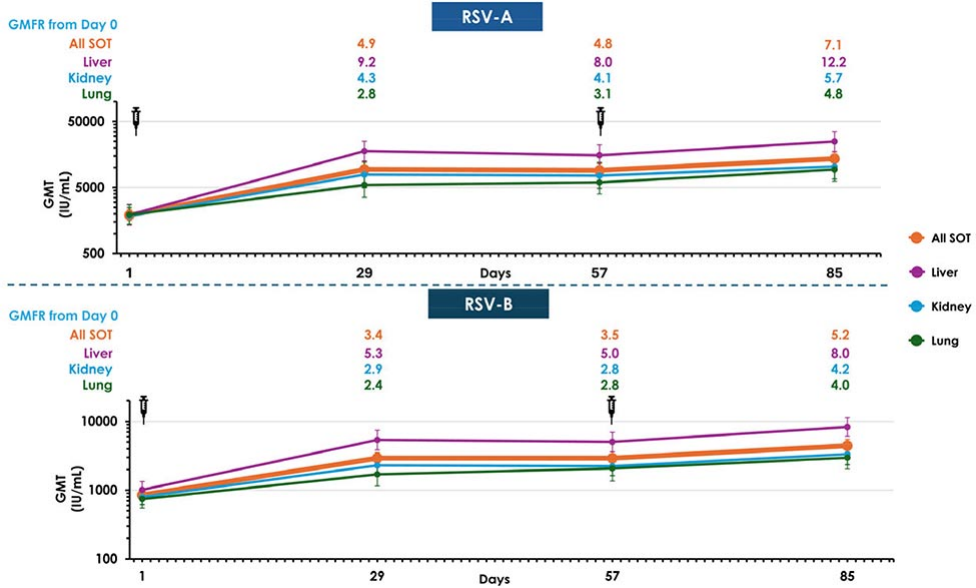
RSV-A and RSV-B Neutralizing Antibody GMTs In Solid Organ Transplant Recipients

**Methods:**

An ongoing, open-label, phase 3 trial (NCT06067230) evaluated 2 mRNA-1345 50-µg doses, 56 days apart, in adults ≥18 years with liver, kidney, or lung transplant ≥180 days prior to the study. Primary objectives included safety, tolerability, and immunogenicity assessed by RSV-A and -B neutralizing antibodies (nAbs). Primary objective included measurement of geometric mean titers (GMTs) on Day 85; secondary objective included measurement of GMTs on Day 29.

**Figure 3.**
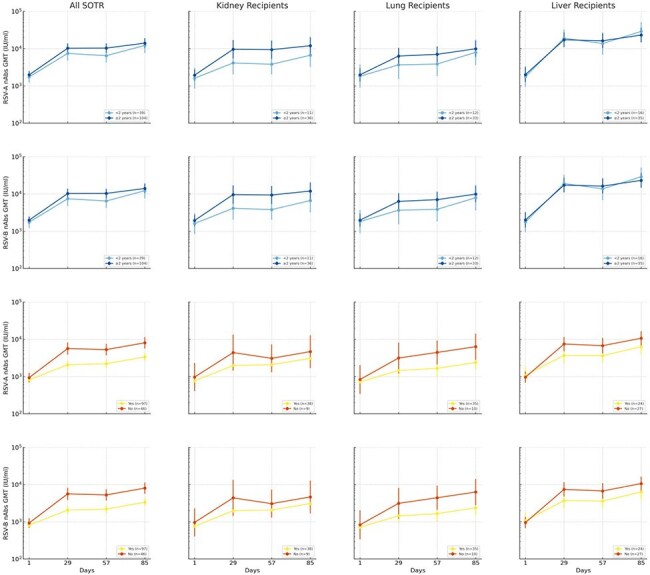
RSV-A and RSV-B Neutralizing Antibody GMTs Based on Time since Transplant (blue curves) and Use of Mycophenolate Mofetil (orange/yellow curves) MMF, mycophenolate mofetil; SOTR, solid organ transplant recipient.

**Results:**

Of 150 SOTR (50 kidney, 52 liver, and 48 lung), 146 received both doses. Median follow-up from Dose 1 was 223 days (range 8-335). Median age was 57 years (range 24–80), 37.3% were female, and 26.7% received SOT < 2 years prior. Most participants (80.6%) were taking concomitant tacrolimus ± mycophenolate ± steroids. Solicited adverse reactions (SARs) within 7 days were similar after both doses (local: 74.0%, 77.4%; systemic: 64.0%, 64.4%; Fig. 1). No grade 4 local SARs, adverse events (AEs) leading to study/vaccine discontinuation, deaths, or AEs of special interest were reported. One month after Dose 1, RSV-A nAbs rose 4.9-fold from baseline; 1 month after Dose 2, nAbs rose 7.1-fold from baseline (Fig. 2). RSV-B nAb response had a 3.4-fold increase from baseline after Dose 1, and a 5.2-fold increase after Dose 2. Titers achieved varied by SOT type. Liver SOTR titers after Dose 1 were comparable to those observed in non-immunocompromised adults in the pivotal efficacy trial . GMTs were lower in kidney and lung SOTRs, SOTRs < 2 years post-transplant, and those on mycophenolate mofetil (MMF), but increased after Dose 2 (Fig. 3).

**Conclusion:**

Two doses of mRNA-1345 50-µg administered 56 days apart in SOTRs were well-tolerated, with no safety concerns. A single dose was immunogenic across all SOTR groups, and a second dose boosted responses, especially in kidney SOTRs, lung SOTRs, those < 2 years post-transplant, or on MMF; therefore, mRNA-1345 is likely to be effective in this vulnerable population.

**Disclosures:**

Dima Kabbani, MD, MSc, AvirPharma Inc.: Grant/Research Support|AvirPharma Inc.: Honoraria for education lectures|F2G: Grant/Research Support|Moderna: Grant/Research Support|Pulmocide Ltd.: Grant/Research Support|Takeda Canada: Consultant fee Deepali Kumar, MD, MSc, FRCPC, Eurofins Viracor: Honoraria|GSK: Advisor/Consultant|GSK: Grant/Research Support|Merck and Company, Inc.: Advisor/Consultant|Moderna, Inc.: Grant/Research Support|Paladin Labs: Honoraria|Takeda Canada: Advisor/Consultant|Takeda Canada: Grant/Research Support

